# Staff goals, challenges, and use of student inquiry in undergraduate bioscience teaching laboratories

**DOI:** 10.1002/2211-5463.13687

**Published:** 2023-08-28

**Authors:** Emily Coyte

**Affiliations:** ^1^ School of Education University of Bristol Bristol UK; ^2^ Learning Science Ltd Bristol UK

**Keywords:** anxiety, bioscience, connection, skills, student inquiry, teaching laboratories

## Abstract

Teaching laboratory practical sessions are major components of undergraduate bioscience curricula, but research into staff perceptions and intentions across institutions in this context is lacking. This study describes a mixed‐methods study using questionnaires and follow‐up interviews to explore staff perceptions of their goals for UK bioscience teaching labs, the extent to which they incorporate student inquiry and challenges encountered with these sessions. The findings show that staff aim for strong lecture connections, applying taught theory to actively develop students' technical and data handling skills. They value teaching labs as opportunities for authentic contact through hands‐on learning with scientific equipment and human connection with staff and other students. Student inquiry (e.g. experimental design decisions) was present in individual elements of teaching labs but not deeply embedded. Staff participants saw teaching labs as first steps to scientific inquiry, often intending to adopt more inquiry activities, but were concerned about time investment and student readiness, especially for early‐year students. Staff who used more inquiry showed stronger goal focus on scientific reasoning, research experience and collaboration. Staff enjoy teaching labs and consider them meaningful learning experiences. Time and budget limitations were more constraining than sense of agency, but overriding challenges were student lab anxieties, and finding ways to increase their confidence and preparation for these sessions. These findings collate staff perceptions of teaching labs across UK institutions and could facilitate reflection, discussion and further research on the goals and impact of these prevalent but resource‐intensive sessions on training the next generation.

## Graphical abstract

Teaching laboratory sessions are unique, ubiquitous yet understudied aspects of bioscience curricula. But what goals do staff have for them? In these active spaces, to what extent do students make experimental decisions (i.e. student inquiry) versus following instructions? What challenges constrain staff goal achievement? Through staff questionnaires and interviews, this study develops a cross‐institutional picture across UK bioscience teaching labs.

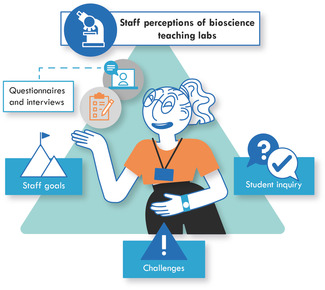

**Abbreviations**
HE: higher education
IQR: interquartile range
QAA: Quality Assurance Agency for Higher Education
RQ: research questions

Video

## Introduction

### Teaching laboratories are unique, ubiquitous yet understudied facets of bioscience curricula

Teaching laboratory sessions have been part of scientific curricula for over a century [1, 2] and remain integral parts of bioscience curricula in higher education (HE). They are recommended and often required by bioscience accreditation and regulatory bodies as means to providing high‐quality skills training in an inclusive, hands‐on way [3, 4]. UK bioscience students spend roughly 3–9 h a week in teaching labs [5], particularly during the first 2 years. Experiments may be performed individually, but more usually in pairs or peer groups [6] supported by academic staff, technicians and demonstrators [7].

Teaching lab sessions are highly resource‐intensive to run, in terms of student and staff time, energy, physical space, costs of reagents and use of equipment [8], yet their presence has become so ubiquitous that their necessity is often given as self‐evident rather than explored on an evidential basis [8, 9, 10], and so they remain understudied relative to other teaching and learning environments [11]. For example, only a quarter of biochemistry educational research literature is based on teaching labs [11]. What does exist largely consists of interventions taken at individual institutions, using student feedback and/or learning outcomes as evidence [see 12, 13, 14 for UK examples]. While these offer valuable insights into what changes are possible, no known research synthesises staff voices across institutions to what their overall perceptions and intentions are for sessions in a UK context.

### Staff goals for teaching labs are important, multifaceted and poorly understood

Despite their ubiquity, there is a longstanding need to define ‘goals’ in the context of teaching labs [8, 10], distinct from experimental aims and learning outcomes [as used by 15, 16]. For this study, staff *goals* for teaching lab sessions are defined as intended high‐level or overarching purposes, usually in terms of student benefit [as used by 17]. While experimental aims and learning outcomes are highly discipline‐specific and bespoke to each session, goals are more generalisable and so can be studied comparatively across UK institutions and bioscience fields.

Within the literature, initial studies to characterise HE teaching lab goals have shown prevalent goals of students mastering lab techniques and developing critical thinking skills in a scientific lab context. [18]. The focus sometimes differed depending on how experienced the students were: foundational units aimed for a stronger connection between lab and lectures, whereas more advanced ones focussed more on experimental design [18].

This work was adapted into a validated survey instrument for determining staff goals for teaching labs [17]. Seven main factors (question groupings found to be connected via statistical methods) were identified: research experience, group work and broader communication skills, error analysis, data collection and analysis, connection between lab and lecture, lab‐specific transferable skills, non‐lab‐specific transferrable skills and lab writing. This instrument's initial focus was US chemistry faculty, a context into which it has been further used and adapted [19], though its application in UK bioscience is not known.

### Teaching labs can offer unique active inquiry opportunities, often unused

It has long been recognised that teaching labs offer unique active inquiry and investigation opportunities for science students [10], making it worth exploring as part of understanding how to get the most from teaching lab sessions. Inquiry (also spelled enquiry) refers to various means by which scientists and students investigate the natural world through questioning, evidence collection and analysis [20]. When applied to education, this commonly involves staff acting more as supporting facilitators than providers of explicit instruction, thereby giving students more control over experimental decisions, design and interpretation [21]. Put another way, it intends to shift students from *learning* about science to *doing* science.

Designing practical sessions around active inquiry has been encouraged to capitalise on this unique potential [22], including in UK bioscience [23]. The Quality Assurance Agency for Higher Education (QAA) subject benchmark statement for bioscience specifies that lab practicals should be inquiry‐driven where possible, including before final‐year projects [3]. Research studies in this context have reported higher student confidence in lab skills [24], more positive attitudes towards authentic research, stronger performance and higher learning gains [25]. This is compared with more didactic ‘cookbook’ experiences where students follow protocols like recipes with predetermined outcomes [26]. This widespread format provides large cohorts some practical experience in a fairly resource‐efficient way, but there are concerns it requires mainly lower‐order procedural thinking [27] over full engagement with the larger purposes of their investigation and the steps required to achieve it [9]. On the contrary, inquiry‐centric sessions involve more complexity and uncertainty by nature, requiring a shifting of roles and requirements for both staff and students [28]. They can be time‐consuming to implement and run [27], and may induce anxiousness, reluctance [29] and a need for additional support [26].

### Incorporating degrees of inquiry into teaching labs may support students at different stages

Inquiry activities have indeed tended to focus on later undergraduate years [30]. Although it has been successfully incorporated into large introductory classes [e.g. 31, 32], others express concerns about student readiness for inquiry. For novice students in particular, more guided instruction may be effective in reducing excessive load on working memory [33]. However, inquiry does not need to be introduced wholesale throughout a course, as measurable skill improvements are possible from adapting even one lab session [34]. Additionally, there is a continuum upon which staff can implement student inquiry into a teaching lab session from asking students to make minor decisions within an otherwise fully instructive protocol to giving them responsibility for a small research investigation [35]. To convert this spectrum into a more categorical rubric to facilitate discussion and comparison, Bruck *et al*. [36] characterised five levels of inquiry (Table 1), extending previous models developed by Schwab [37] and Herron [38]. This was further adapted by Brownell & Kloser [39] to show whether the experimental outcome is known to the teaching staff, indicative of procedural cookbook format.

**Table 1:** Rubric of levels of inquiry in undergraduate laboratories, adapted from [36, 39].

Level	Given name	Provided for students	Designed/produced by students	Known outcome
0	Confirmation	Problem, procedures, method of analysis, results and interpretation	n/a	Yes
½	Structured inquiry	Problem, procedures and method of analysis	Results and interpretation	Yes
1	Guided inquiry	Problem and procedures	Method of analysis, results and interpretation	Yes
2	Open inquiry	Problem	Procedures, method of analysis, results and interpretation	No
3	Authentic inquiry	n/a	Problem, procedures, method of analysis, results and interpretation	No

Under this rubric, inquiry level is determined by the number of experimental stages where student decision and production is required. This categorisation has been used by others [e.g. 40, 41] and is adapted here for discussing the dimensions by which student inquiry can be incorporated.

### Budgets, time, agency and sense of meaningfulness may limit the realisation of teaching lab goals

Faculty goals do not always align with the actual experiments run [42]. Whether the intentions staff have for teaching labs translate into actions may be constrained by limiting factors, such as budget, agency, time or sense of meaningfulness.

In recent decades, UK HE has undergone massification [43] as student numbers in UK HE persistently rise [44], increasing demand across the country. However, institutional budgets are stretched and governmental support is often uncertain [45]. HE staff have relatively high levels of autonomy at work [46]; however, the prevalence of role ambiguity and conflicting requirements can reduce job satisfaction [47]. Furthermore, the majority of UK HE staff find their jobs highly stressful and work long hours (three‐quarters work more than 40 h a week, and over a third work more than 50) to keep on top of competing demands [46]. Opportunities for reflecting and changing teaching lab sessions may therefore be limited.

Meaningful learning is essential for conceptual change, allowing students to effectively integrate new knowledge to their own cognitive structure [48]. Teaching staff report more negative perceptions of teaching labs' potential for meaningful learning than students do [49], but it is not known whether this limits decisions about what and how to teach in these sessions.

### Skill disparities, anxiety and lockdowns impact student experiences of teaching labs

Student journeys leading up to university are increasingly varied as the student body becomes more diverse [50]. While wider HE access should be welcomed, staff are now teaching cohorts with greater disparity of skills, increasing the challenge of ensuring all needs are met [51]. This applies to teaching labs in particular, which put high cognitive demands on students as they navigate a new physical environment, manipulate equipment and data as well as getting to grips with underlying theoretical concepts [13].

For many students, labs can be a source of anxiety [52], especially as the frequency of science practicals in UK schools continues to decline [53], and international students' prior experiences are highly varied. Practical teaching was particularly affected when this study was conducted in Spring 2021, amidst the COVID‐19 pandemic and subsequent lockdowns. Staff needed to rapidly shift to online alternatives, defer or cancel labs [54], and impacts rippled through the years as each ‘COVID cohort’ had uniquely disrupted learning, assessment and social experiences [55]. Minimal practical experience means students arrive lacking in lab confidence, inhibiting their enjoyment and learning, although this can be mitigated by pre‐lab preparatory activities [12].

### Research framework

**Fig. 1:**
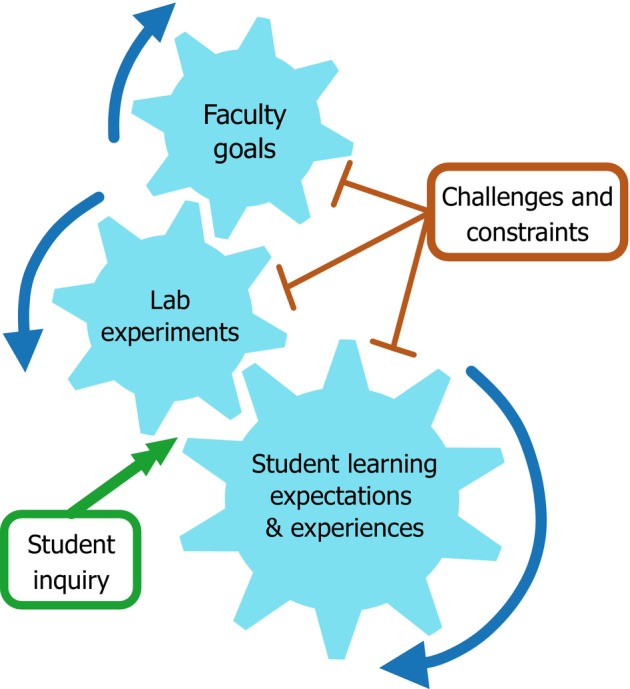
Model of how staff goals drive lab experiments and student learning experiences based on [42]. Double‐headed arrow of inquiry represents additional student‐driven motion, and flatheads represent potential inhibition.

Staff goals for teaching labs both influence and drive the type of experiments run in teaching labs, in terms of both experimental topic and format. This in turn influences and drives student experiences and expectations for lab learning [42] (Fig. 1). Within this framework, adding elements of inquiry would mean giving students some control over the system's movement, rather than having it be entirely staff‐driven. Additionally, we cannot expect the system to always run smoothly; teaching lab challenges and constraints could be seen as friction, slowing or stalling the system at any given point.

### Study aim and contributions

This research aims to enhance understanding of the unique potential of teaching labs. It provides a novel contribution to the field by initiating a cross‐institutional picture of the perceptions and intentions UK university staff have for their bioscience teaching labs, with specific foci on goals, student inquiry and challenges. This builds upon previous research described earlier, but the combination and adaptation into UK bioscience contexts is not known in the literature.

In achieving these aims via the research questions below, this study can help discussions of teaching labs move beyond self‐evident ubiquity [8] to more explicit articulation, with a goal of enabling more evidence‐based reforms [35] and more learning potential for students.

### Research questions

To achieve the study aims, the research questions (RQs) for this study are as follows:

*RQ1: What goals for students do UK academic staff have for the undergraduate bioscience teaching lab sessions that they manage?*
*RQ2: How is student inquiry perceived and used in these contexts?*
*RQ3: What challenges or constraints do staff encounter in these contexts?*

## Methodology and research methods

### A pragmatic, mixed‐methods research design

These research questions are each addressed using a pragmatic mixed‐methods approach [56, 57], synergistically combining quantitative breadth with the rich depth of qualitative study [58]. Specifically, the study has a sequential explanatory design [59, 60] where knowledge acquired from a primarily quantitative questionnaire (with a minor qualitative element) is used to inform subsequent semistructured interviews [61]. This research was granted ethics approval from the University of Bristol (review reference number 2021‐8313‐8260).

### Questionnaire: recruitment, format and analysis

The questionnaire was created in Qualtrics and deployed for 5 weeks in April–May 2021. The full questionnaire schedule is provided in the Supporting Information. Participation was anonymous and optional, and recruitment took place via direct emails and social media. The sampling frame consisted of staff at UK universities who had organised and/or ran at least one undergraduate bioscience teaching lab practical for at least 2 years, to ensure prepandemic experience. Potential participants were identified from known contacts and through a systematic search of UK university websites (minimum one per institution). Social media posts were made to further raise awareness and encourage participation. Informed consent was obtained upfront via pre‐questionnaire information and checkboxes.

Upon starting, staff participants were asked to select the student year group they work with most regarding teaching labs. As students tackle increasingly complex and demanding lab activities throughout their degree [23], staff who teach multiple‐year groups could have different goals, challenges and use of inquiry in their teaching lab sessions.

The first core questionnaire section included items (i.e. questions) derived from the ‘Faculty Goals for Undergraduate Chemistry Laboratory Survey’ [17]. This survey instrument showed high internal consistency with Cronbach's alpha of 0.904 (*N* = 312) [62] and had face and construct validity improved via piloting [63]. Fourteen of the 29 items were selected to reduce the overall length and encourage survey recruitment and completion, given limited time often available to teaching staff. At least one item from each of the seven factors (listed earlier) was selected to maximise coverage, while removing items more exclusive to chemistry such as error analysis. Example items used include ‘My lab sessions are a place for students to learn to analyse data’ and ‘My lab sessions are designed to foster an appreciation for science in students’, with staff rating 0–100% agreement. Items were randomised to remove systematic biases from respondent fatigue or the impact of previously answered question [64, 65]. One attention check item was included [66], instructing the respondent to select 40% exactly.

An optional free‐text question from the original Faculty Goals survey was also included [17]: ‘What additional information would you offer about goals for your teaching lab sessions?’. Here, participants could provide further detail about their questionnaire responses or introduce new aspects which could benefit from further exploration in qualitative interviews.

Level of student inquiry in each component of teaching lab sessions was addressed in six Likert‐scale questions. These were based on categories identified by Bruck et al [36], including problem, procedure, methods of analysis and interpretation, for example ‘Students have a choice of experimental procedures they want to use in a lab session’, plus one item from Brownell & Kloser [39]: ‘I know what the experimental outcomes of laboratory sessions will be’. Specific mention of ‘inquiry’ was avoided to make it accessible to participants regardless of familiarity with this term. These were answerable on a 5‐point scale using percentage ranges, for example ‘26‐50% of the time’.

To begin exploring challenges and limiting factors, staff were asked whether they enjoyed teaching labs and considered them meaningful learning experiences [48], and whether they thought their students and colleagues agreed for comparison. They were also asked whether they felt they had sufficient budget, agency and/or time to make teaching lab decisions, to determine whether any of these practical constraints were present.

Finally, demographic‐type optional questions included teaching/research role focus, taught bioscience topics, full‐ or part‐time status, gender and years spent running teaching labs. These provided an overview of the respondent group composition and assisted in interview sampling. Participants could opt‐in to be contacted for interview by entering their email address before submission.

Quantitative analysis was performed in SPSS. Distributional tests to determine normality and subsequent test type were performed throughout [67], as were reliability estimates and interitem correlations where relevant. Goal item ratings were investigated to determine how much each was valued by staff participants. Responses to each inquiry item were used to determine whether and how deeply inquiry is embedded in bioscience teaching labs and were summed into a scale for further investigation. Whether enjoyment, sense of lab meaningfulness, budget, time or sense of agency were limiting was considered. Tests for association between goals, inquiry scale, affective and limiting component items and main year groups were also performed. The free‐text responses were analysed in NVivo using summative content analysis for broad information gathering, exploring the prevalence of terms and concepts across participants [68].

### Semistructured interviews: recruitment, format and analysis

Of 79 valid questionnaire responses, 35 agreed to be contacted for interview (44%). This provided an opportunity for purposive sampling [69]. A criteria‐based shortlist was created, including those who ran multiple labs with multiple‐year groups but selected Year 1 or Year 2 as their main cohort, who volunteered information to the free‐text response question as a proxy for willingness to communicate further ideas and who were not personally known to the researcher through other channels to minimise potential role conflict [70] and increase the likelihood of eliciting more detailed responses due to less shared or presumed knowledge.

From the shortlisted 16, eight respondents were selected based on maximum variation sampling [71] aiming for a broad heterogeneity of inquiry levels (as determined categorically by splitting inquiry score into terciles), main year group, institution type, gender and time spent working in teaching labs. Six responded to follow‐up communication, and the resulting data were deemed sufficiently rich for thematic analysis [72]. Interviews were conducted online via Zoom in May–June 2021.

The semistructured format was chosen for its versatility at the intersection between structure and fluidity. Each interview was guided by a loose question schedule covering core topics derived from collective and individual questionnaire responses, with opportunities for elaborations and unique focal areas within that [73]. Using questionnaire responses offered a head‐start to discussion, meaning interviews could be kept reasonably brief to encourage participation, anticipating the busy schedules of academic staff (Mean = 37 min).

Initial interview transcripts were auto‐generated by Zoom and then edited for accuracy by the researcher shortly after the interview [73]. The transcripts were coded inductively in NVivo [74] and qualitatively interpreted using reflexive thematic analysis. This approach was chosen for its full committal to the qualitative strengths of rich data and human connection, providing depth to support the quantitative questionnaire. Reflexive thematic analysis is named for its acknowledgement of the researcher's active role and decisions taken throughout the process of transcribing, coding and generating themes [75]. Under reflexive thematic analysis, themes are not simple domain summaries or ‘buckets’ of data which passively emerge from the data. Instead, they are patterns of shared meaning underpinned by central organising concepts, cutting across individual points [75], generated by the researcher through active immersion among the data [76] [77].

## Results

### Questionnaire participant demographic characteristics

The questionnaire method was chosen to establish a broad snapshot of UK staff perceptions and intentions regarding bioscience teaching labs, to help maximise the unique potential of these sessions. Of 81 survey responses, 79 were included for analysis, as the remaining two failed the attention check item. There were similar numbers of female and male respondents (49% vs 46%), and roles were largely split between primarily teaching (47%), and combined research and teaching (44%). Most worked full‐time (90%), and the majority had been running or managing teaching labs for over 5 years (79%). See Table S1 for more detail. Of bioscience topics taught, there was the highest representation from cell and molecular biology, biochemistry and microbiology. However, each listed bioscience topic was selected by at least 9% of participants, and no topic had more than 42% coverage, showing satisfactory diversity across the field.

Most respondents (86%) ran teaching lab sessions for multiple‐year groups. When asked to select which teaching lab year group they worked with the most, there was a close split between Year 1 (42%) and Year 2 (43%), with Year 3 making up the remaining minority. Most (89%) run multiple different lab sessions for their chosen year group, with 4–9 labs being the most common response (34%). See Table S2 for more detail.

### Surveyed staff goals: reliability, intercorrelations and distribution information

**Fig. 2:**
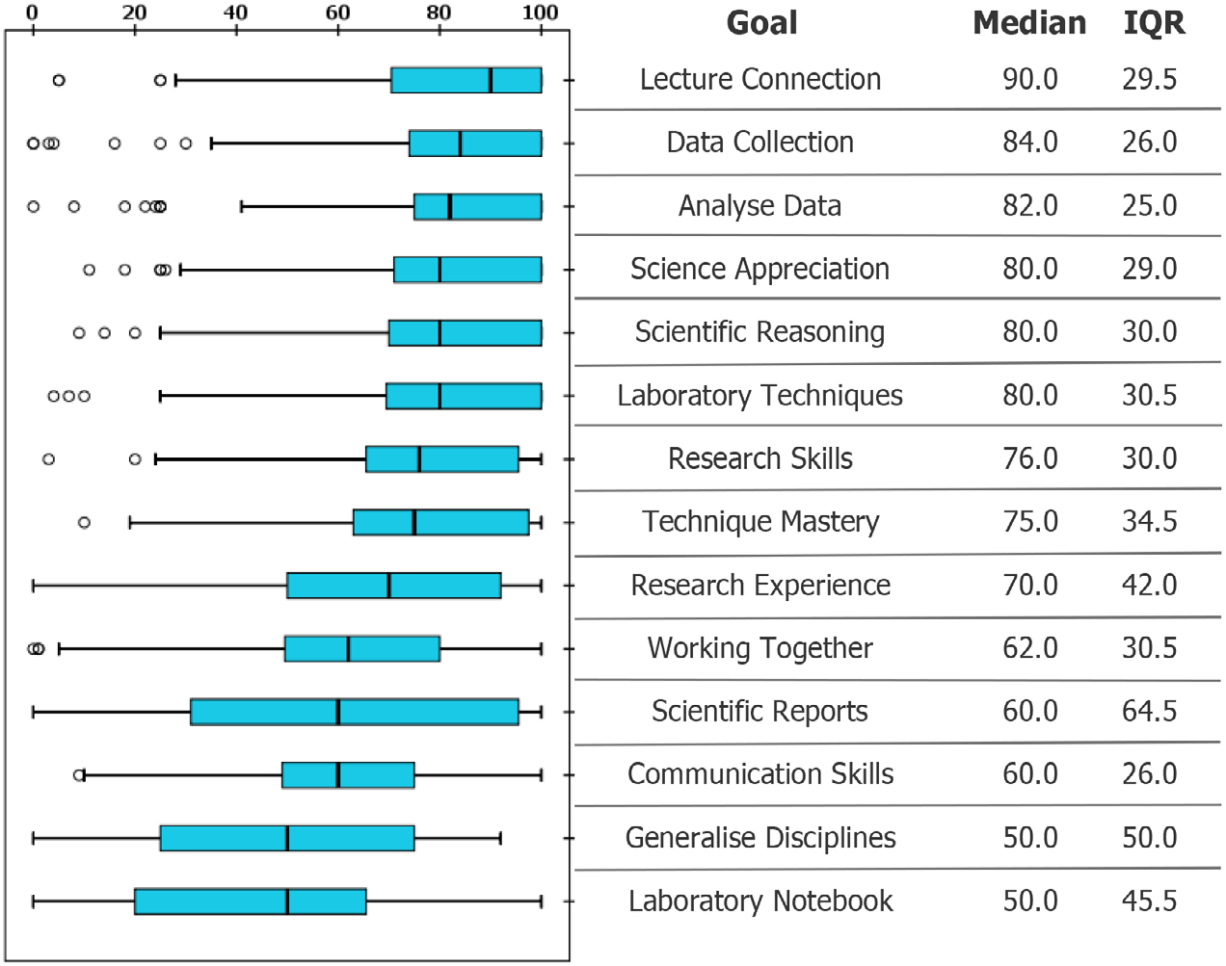
Staff (*N* = 79) ratings of 14 teaching laboratory goals derived from [17], sorted by median. Boxes show medians and interquartile ranges (IQR) using Tukey's hinges. Whiskers show range, excluding outliers (represented by circles).

Staff participants were asked to rate 14 teaching lab goal items (derived from [17]). Goal ratings were not normally distributed (Shapiro–Wilk *P* ≤ 0.026). Therefore, with the context of a modest sample size, ratings are presented as box plots (Fig. 2), described with medians and interquartile ranges (IQR) rather than means and analysed using nonparametric tests [67] such as Mann–Whitney for intergroup differences and Kendall's tau for correlations.

The median rating across all goals combined was 75 out of 100, suggesting that goal items generally resonated with staff. The responses showed good reliability, with Cronbach's alpha of 0.845 [62]. Goal responses were broadly but not intensely correlated (see Table S3 for the correlation matrix), but no correlation was excessively high. This indicates there was sufficient distinction between items, so none were considered for exclusion.

### Prevalent staff goals include lecture connection, data collection and analysis

The highest scoring goal was about strong connection between lecture content and lab sessions (Median = 90, IQR = 29.5), followed by goals relating to data collection techniques (Median = 84, IQR = 26.0), and learning to analyse data (Median = 82, IQR = 25.0; Fig. 2). Although not exactly unpopular options with medians of 50 out of 100, a joint‐lowest ranked goal was generalising to multiple disciplines (IQR = 50.0) and keeping good lab notebooks (IQR = 45.5).

### Goals for Year 1 vs Year 2 teaching labs are not statistically different

Two‐tailed Mann–Whitney tests were used to determine whether academic goals differed between Year 1 (*N* = 33) and Year 2 (*N* = 34) groups. The Year 3+ group were excluded due to the much smaller sample size (*N* = 12) and variability of final‐year lab experiences. The lecture connection rating of Year 1 labs (Median = 95, IQR = 20.0, *Z* = 1.9) was much higher than Year 2 (Median = 76, IQR = 33.0); however, this did not reach statistical significance, *U* = 415.0, *P* = 0.062. Similarly, the goal for generalising across disciplines was higher for Year 1 (Median = 60, IQR = 30.0, *Z* = 1.9) than Year 2 (Median = 50, IQR = 25.0), yet this difference was also not statistically significant, *U* = 410.0, *P* = 0.058. None of the other items showed statistically significant differences (*P* > 0.2).

### Low‐to‐modest use of student inquiry across bioscience teaching labs

**Fig. 3:**
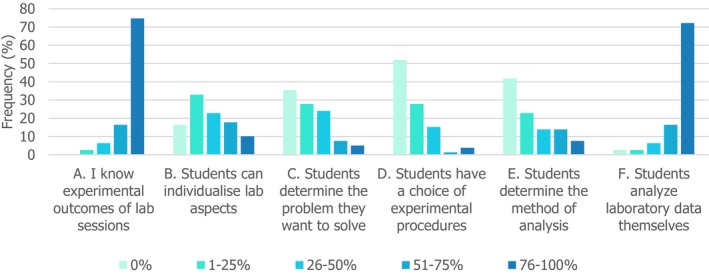
Staff reports on percentage of their teaching lab sessions which (A) have previously known experimental outcomes and (B–F) require students to make decisions in different stages of the session.

A unique aspect of teaching labs is the unique opportunities for student active inquiry [10], which has been reported as conducive to positive outcomes [24, 25], but can be challenging to implement [28]. Staff were asked about the percentage of labs in which students must make decisions in different parts of the lab experience, in alignment with adapted literature rubric levels [36, 39]. Staff reported an overall low‐to‐modest use of student inquiry (Fig. 3). Most (75%) said they knew the expected experimental outcomes of their lab sessions 76–100% of the time, indicating procedural cookbook labs with low inquiry. A further 17% said they knew 51–75% of the time, indicating occasional use of inquiry. There was commonly *some* element of choice with 56% of respondents giving students opportunities to individualise aspects of their experiment 1–50% of the time.

Breaking experimental stages down into components, it was very common (89%) for students to be asked to analyse and interpret their own data in at least half of their labs. Other components were less prominent: choice of analysis method featured in fewer than a quarter of labs for 65% of respondents, and students had to determine the problem they want to solve in fewer than a quarter of labs for 63% of respondents. It was rarest for students to have choice of experimental procedure, with most (80%) offering it in less than a quarter of labs, and over half (52%) of staff never providing it.

### Staff who incorporate more student inquiry scored certain lab goals more highly

A combined inquiry scale was created by summing the six items each on a 5‐point scale, with ‘0% of the time’ scoring 0, and ‘75–100% of the time’ scoring 4 [17]. A higher score on this scale (ranging from 0 to 24) was taken as a proxy for deeper implementation of inquiry‐based learning. Some inquiry items showed intercorrelations (See Table S4), but none above Kendall's τ = 0.443, so no items were considered for exclusion. Item 1 (knowing experimental outcomes) was reverse‐coded due to the wording. The resultant inquiry scores (Median = 8.0, IQR = 5.0, *N* = 79) had an acceptably high Cronbach's alpha at 0.705 [62] but showed statistical deviation from normality (Shapiro–Wilk *P* = 0.005). Therefore, nonparametric tests are used here.

Participants showing stronger use of inquiry in their teaching labs rated six of the goal items significantly higher, in particular scientific reasoning (Spearman's ρ = 0.399) and keeping a lab notebook (ρ = 0.295) were highly significant, with *P* < 0.01. Goals for research experience, communication skills, working together and science appreciation were also rated significantly higher in those with higher inquiry scores, with *P* < 0.05. No other goals showed a significant correlation with inquiry score, positive or negative.

Combined inquiry score was higher for staff focussing on Year 2 (Mean Rank = 37.2) compared with Year 1 (30.7); however, this was not statistically significant (two‐tailed Mann–Whitney *U* = 452.5, *P* = 0.174, *Z* = 1.4). Investigating further with individual inquiry items: in Year 2 labs, students are expected to individualise aspects of their experiment significantly more than in Year 1 (Mean Rank = 38.8 vs 29.0, *U* = 396.5, *P* = 0.033, *Z* = 2.1). No other item was statistically significant, but each was higher for Year 2 than Year 1.

### Teaching labs are considered meaningful and enjoyable, although budget, time and agency may be limiting

**Fig. 4:**
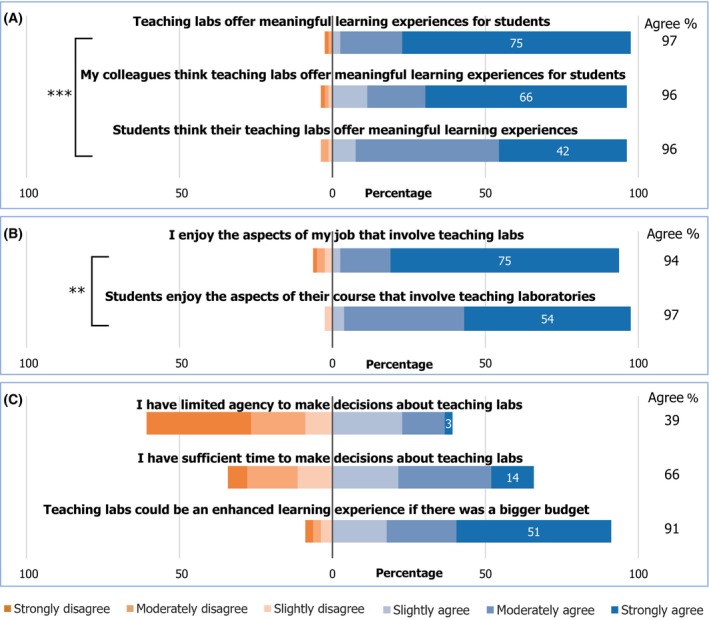
Agreement to statements about potential challenges and limiting factors of teaching lab decisions, including perceptions of (A) meaningful learning, (B) enjoyment and (C) sense of agency, time and budget around teaching labs. Wilcoxon signed‐rank: **P < 0.01, ***P < 0.001.

Bioscience staff respondents consider teaching labs to be meaningful learning experiences and believe their colleagues share this view. They also believe their students consider teaching labs meaningful learning experiences, but the response was more muted and significantly lower as determined by Wilcoxon signed‐rank, a nonparametric paired test of difference (*Z* = −3.8, *P* < 0.001 exact; Fig. 4A). Most respondents enjoy aspects of their role involving teaching labs, and many believe their students enjoy them, though again to a significantly reduced extent (*Z* = −2.7, *P* = 0.008; Fig. 4B).

Staff views on limited agency showed a bimodal split; the most common was ‘strongly disagree’ (34%) followed by ‘slightly agree’ (23%) (Fig. 4C). Views were similarly mixed about whether there was sufficient time to make teaching labs decisions, although more participants moderately or strongly agreed (44%) than disagreed to the same extent (23%). There was however broad agreement that bigger budget would enhance labs' learning experience (73% strongly or moderately agree).

Staff who more strongly agreed that teaching labs would benefit from a bigger budget also more strongly agreed that they had limited agency (Kendall's τ = 0.265, *P* = 0.005). Feelings of sufficient time were negatively but not quite significantly correlated with feelings of limited agency and budget (τ = −0.160, *P* = 0.080 and τ = −0.167, *P* = 0.076, respectively). Those with sufficient time are more likely to enjoy teaching labs (τ = 0.264, *P* = 0.007), but agency or budget limitations were uncorrelated with enjoyment (*P* > 0.5). Neither belief in teaching labs' meaningfulness nor perceptions of student enjoyment were associated with sense of agency, time available or budgetary concerns (*P* > 0.17). See Table S5 for the correlation matrix. Perceptions of meaningful learning, enjoyment or the three constraints discussed were not significantly associated with the use of inquiry (Kendall's tau, *P* > 0.13) or when comparing Years 1 and 2 labs (Mann–Whitney, *P* > 0.28).

### Questionnaire free‐text content analysis highlighted a focus on skill development, with preparatory and affective aspects

**Fig. 5:**
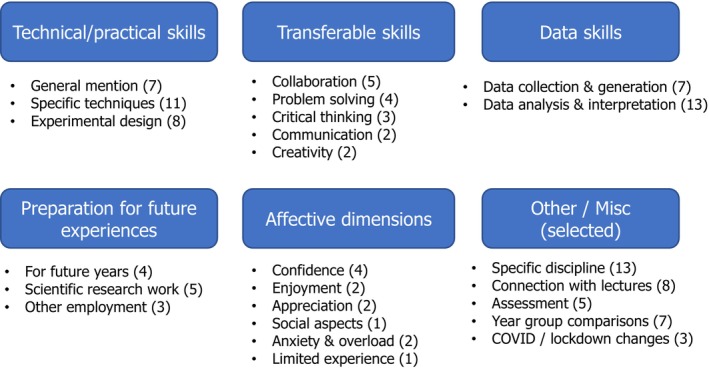
Content analysis of responses (*N* = 43) to the free‐text question ‘What additional information would you offer about goals for your teaching laboratory sessions?’, showing selected categories and subcategories. Bracketed values show number of staff responses.

Content analysis on the 43 responses to the free‐text question ‘What additional information would you offer about goals for your teaching laboratory sessions?’ (Fig. 5) gave further insight into staff perceptions and provided suggestions for consideration in the follow‐up interviews.

The responses showed prevalent emphasis on a wide variety of skills, including technical and practical, data and transferable skills. There was also a notion of teaching labs introducing genuine lab experiences and preparing students for more complex research environments, such as final‐year projects, and working in research labs beyond that, for those who choose that path.

No participant mentioned ‘inquiry’ specifically; however, experimental design was mentioned or described fairly frequently. Among those who discussed progression or made year group comparisons, references to Year 1 labs tended to mention fundamentals, whereas Years 2 and/or 3 were associated with research and experimental design, suggesting more targeted aspects for discussion in the interviews.

References to COVID‐19 impacts were surprisingly low given its impact on education at the time [78]. Rather more prevalent were mentions of the affective aspects of labs. Some were framed positively around developing confidence and enjoyment, but others mentioned anxiety or cognitive overload about lab situations, and some students having limited lab experience prior to university. These combined findings hinted at potentially fruitful topics for interview exploration.

### Interview participant characteristics

The interview method was chosen to develop richer insight into participants' individual lived experiences and attitudes towards their teaching lab sessions [79]. As previously described, 35 out of 79 questionnaire participants (44%) agreed to be contacted for an interview. This group showed no significant difference (*P* > 0.05) compared with those who did not provide contact details in terms of goal ratings, inquiry score or perceptions of enjoyment, meaningful learning or budget. However, perhaps counter‐intuitively, those who agreed to be contacted for an interview had significantly more ‘limited agency’ and less ‘sufficient time’ (Mann–Whitney *P* = 0.003 and *P* = 0.041, respectively). Largely though, the interviewee pool was representative in terms of areas relevant to the research questions.

Six staff interviewees were purposively sampled from those who volunteered from questionnaire participation. All ran multiple labs with multiple‐year groups for comparison opportunities, but had a broad heterogeneity of other characteristics (Table 2). These participants had different institutional contexts, but their perceptions of bioscience teaching labs were sufficiently comparable that themes could be generated which further illuminated questionnaire findings or added new dimensions. As previously described, these themes are not claiming to be ‘definitive’, for example goals or challenges (for example) of teaching labs. Instead, they are more like multifaceted currents which were identified as flowing throughout the interviews, either at the surface or just beneath.

**Table 2:** Selected interviewee characteristics showing heterogeneity and similarities. Pseudonyms are gender‐matched common UK names to preserve anonymity.

Pseudonym	Institution type	Main year group	Num. sessions for main year group	Years teaching lab experience	Role type	Relative use of inquiry
Emma	Post‐1992	Year 1	4–9 lab practicals	2–3 years	Full‐time, teaching	Medium
Simon	Post‐1992	Year 1	4–9 lab practicals	6–10 years	Full‐time, teaching	Medium
Daniel	Russell Group	Year 2	10+ lab practicals	2–3 years	Full‐time, teaching	Medium
Adam	Pre‐1992	Year 1	10+ lab practicals	11+ years	Full‐time, teaching	High
Claire	Russell Group	Year 2	2–3 lab practicals	11+ years	Full‐time, teaching	High
Rich	Russell Group	Year 2	2–3 lab practicals	6–10 years	Full‐time, teaching and research	Medium

### Theme A: Teaching labs as places of physical and social contact

#### Student‐to‐equipment contact

Teaching labs were frequently described as places for hands‐on learning. This is connected with active learning (see Theme B), but there was a distinctly physical, tangible aspect associated with being present in the lab. Participants considered this aspect enjoyable to students and important for learning. These interviews took place in late spring 2021, and the implications of COVID‐19 lockdowns and restrictions for teaching labs were keenly felt by staff.

‘There's a lot of learning opportunities that they've missed because they've not being able to come in and see things hands‐on, to move things around, get that 3D appreciation’.Daniel

#### Student‐to‐staff contact

A more affective, human aspect of this theme is the otherwise‐rare social contact opportunities that teaching labs provide. Staff valued being able to circulate during sessions, spot issues and address misconceptions, offer help or answer questions where needed. These teaching lab interactions were also viewed as opportunities for staff and students to connect and get to know each other as individuals. This appears to be more of a side‐effect than an intended purpose of teaching labs, but a highly valued one within a largely online educational system.

‘It's my main chance to get to know the students really, and to get to learn their names’.Adam

‘In the labs you get that chance to go round and talk to people individually, help them and guide them through stuff’.Simon

#### Student‐to‐student contact

Staff appreciated that teaching labs afforded students the chance to spend social time with their peers and work together on a shared goal. Sometimes pair and group work could create tensions if there were disparities in ability or motivation, but overall working together gives the labs a ‘lived‐in’ feel, leading to a collegial sense of mutual aid which can grow over consecutive sessions.

‘There's the social thing, because they're working typically in pairs, they get to talk about the work or about other things of course, so that helps to build community’.Adam

In the main, every participant brought up the contact students get with equipment, staff and/or other students as a valued, even precious, component of their bioscience curricula.

### Theme B: Teaching labs as a site for actively developing skills as applications of theory

#### Active learning and skill development

There was a broad and prevalent perception of teaching labs as experiential places of action and activity for students. This was seen as is important for learning and development, as well as exciting and giving a sense of satisfaction and achievement.

‘It's exactly why practical learning is so pedagogically important, because it really is. That's where our students do so much of their active learning: in the labs’.Adam

#### Application of lecture connection

Mirroring the questionnaire findings, labs and lectures were seen as closely connected. Exploring this relationship a little further, lectures had a foundational or preparatory role to provide students with theoretical underpinnings to teaching lab sessions. Teaching lab sessions were often deliberately timetabled close to lectures on relevant topics and were intended for students to apply lecture content in practical settings, bringing life to taught theoretical content.

‘…putting everything into context and applying the knowledge they learned in lectures to in the labs’.Emma

#### Skills for research and the future

Similar to the content analysis findings, the skills discussed largely included specific techniques (e.g. pipetting, microscopy and dissection), as well as data collection, handling and analysis skills. Transferrable skills were discussed more implicitly, possibly because these topics operate more at whole‐year or degree‐level than individual sessions. Research aspects were prominent, and there were hopes that students would take these forward in future experiences. Sometimes this is made explicit to the student, other times it is expected to develop naturally throughout the year's sessions.

[We say to students:] ‘Right, these are the things you're studying, these are the things they can actually be used for, this is how you apply them in a research setting.’Claire

### Theme C: Teaching labs have the potential to be tentative first steps of student inquiry

The term ‘inquiry’ was used just once, perhaps indicating that this term is not prevalent in practitioner discourse of teaching labs. Instead, discussion generally proceeded in terms of ‘experimental design’ as seen in the questionnaire free‐text, or student decisions before or within the lab, or hypothesis‐driven activities. Where inquiry elements were used, it was usually individual aspects of choice within instructor‐defined bounds.

#### Timings within the university experience

Staff recognise that students enter university with a wide skill disparity (see also Theme D). Participants expressed a general sense of students not being ready for more experimental design decisions in Year 1. Instead, Year 2 was viewed as an optimal time for introducing these aspects, for example in preparation for larger third‐year projects.

‘So Year One, I can say is very much the basic scientific concepts … Second year is bringing in more professional skills, bringing in some more of that experimental design’.Emma

#### Familiar but frustrating cookbook labs

The most common descriptions of participants' own labs involved following protocols, possibly with some (usually limited) element of choice. Yet, there were frustrations and even somewhat dismissive language around this cookbook format, indicating tacit or explicit acknowledgment of some of its limitations.

‘Rather than just giving them a recipe to have to follow…’Adam

[speaking of a previous course's labs] ‘Some of those were very much this: ‘Follow 10 steps and you're done’ kind of thing. So the inquiry‐based approach is not there so much’.Rich

#### Inquiry intentions vs tensions

In addition to concerns of student readiness, some (but not all) interviewees said they hoped or intended to incorporate more experimental design decisions into their labs, but had not gotten around to it due to concerns of time constraints or student perceptions. There were tensions between having time‐efficient, reliable (cookbook) experiments most students will satisfactorily complete versus the freedom and discovery of more inquiry‐led work.

‘It's something that we want to bring more into the first and second year… it's giving them more choice in what they do, and more space to go wrong, explore and experiment’.Simon

‘I think [students] want things to be bulletproof on the one hand, but I think they like the discovery … I think that's harder to do in the context of a three hour practical’.Rich

### Theme D: Alleviating student anxieties and hesitancies to engage with teaching labs

The interviews largely mirrored the questionnaire responses to budget, time or agency challenges in terms of how mixed they were. Although there were isolated mentions of aspects like physical space issues, and an underlying current of being spread too thin with multiple commitments, these came up less than expected.

#### Lab anxieties

Instead, a challenge which came up in every interview was student anxiety, nerves and low confidence in teaching lab settings. This can reduce student engagement with labs to the point they do not show up. There was no blame or dismissal of students' feelings; rather, compassion and concern that this persists, even worsening with each cohort.

‘As a general observation, I'm finding students are increasingly afraid to do something, because they might get it wrong, so they're afraid to fail’.Rich

‘I think a lot of people are very anxious about coming into lab sessions and so decide not to come’.Emma

#### Experience disparity and inclusivity

There was some acknowledgement that students have a wide disparity of lab experiences prior to arriving at university, maybe none at all. Students were also arriving with a range of additional needs, adding an inclusivity angle.

‘We've got students from variable backgrounds and about 30% will have a learning support plan for either physical or mental health related disability, or something else that they may not disclose’.Claire

‘Everybody has different skill levels to start with. Some of our students have never been in a lab before at all, in their lives’.Simon

#### Lack of student preparedness

Student engagement within the lab itself was never raised as an issue, students being ill‐prepared for the lab through not engaging with assigned pre‐lab materials was often flagged. Acknowledging this was true for a ‘significant minority’ rather than universal, it was a continued challenge and regular source of frustration for many interviewees.

‘They're not always as prepared as we'd hoped that they would be, which does make it a lot more difficult when you actually get into the lab’.Daniel

#### Handling with communication

The way that these are handled appears to be a combination of providing more and different types of preparatory resources to demonstrate as clearly as possible what they can expect. Online learning has a role to play here, allowing students to asynchronously prepare. However, students still not engaging with these was a continued source of frustration.

‘The students who might experience anxiety being in a lab situation, they said they found it much better being able to see in advance what they would be doing. Because a lot of the anxiety for some of them comes from the unknown, not knowing the expectations’.Claire

#### Handling with connection

As described in Theme A, student contact was a major part of teaching labs, and this is reflected here too. Another way of alleviating student anxieties was through reassurance and establishing dialogues with students, making sure they know there are communication channels available.

‘It's reassuring them that they're here to learn these skills and we don't expect them to come with these skills’.Simon

‘[We] encourage them to talk to each other, encourage them to talk to us if there are aspects of anxiety that are affecting them’.Daniel

### Thematic map

**Fig. 6:**
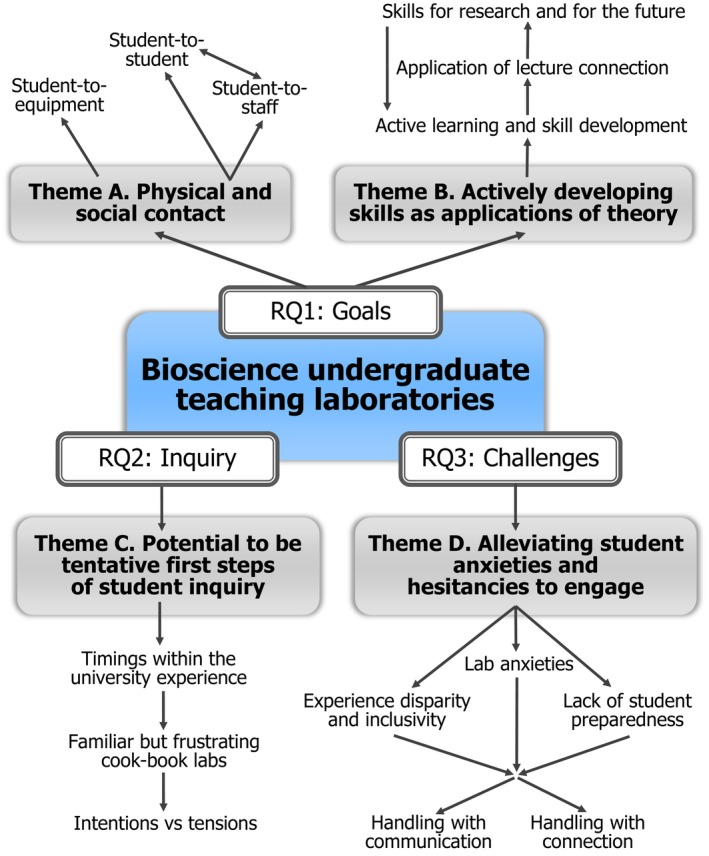
Thematic overview of interview analysis, showing four themes generated around three research questions (RQs), subthemes and loose representation of their relationships.

Figure 6 provides an overview of the reflexive thematic analysis findings.

## Discussion

This mixed‐methods study explored staff perceptions of the UK bioscience undergraduate teaching lab sessions they run or manage, particularly in relation to their goals for these sessions, how they perceive and use student inquiry, and the challenges and constraints they encounter. This is the first known study to synthesise staff perceptions of these topics in a UK bioscience context. Data were produced and analysed from 79 questionnaire responses and six follow‐up interviews.

The study addressed three research questions (RQs), listed previously. Considering staff goals for teaching labs (RQ1), these sessions were seen as places for tangible connection with lectures, physical equipment, staff and other students. Prevalent goals also included skill development in areas such as data collection and analysis. Student inquiry (RQ2) was used sparingly and cautiously, generally from Year 2 onwards. Teaching staff recognised the active learning benefits while acknowledging time constraints with setting up inquiry‐rich sessions. Key challenges (RQ3) included student anxieties and hesitancies to engage with pre‐lab and in‐lab activities. Budgetary constraints were fairly common, more so than sense‐of‐agency or time constraints. Teaching staff still enjoy teaching labs and consider them meaningful. The remainder of this section will discuss the findings from each research question in more detail.

### RQ1: Staff goals for bioscience teaching labs

Bioscience teaching staff participants provided rating for 14 teaching lab goal items (derived from [17]). The findings here showed goals were particularly focussed on lecture connection, data handling and scientific reasoning and appreciation. The top rating of lecture connection suggests teaching labs are being considered as part of a wider whole within the taught unit, which is likely conducive to learning as coherent alignment is recommended in curriculum design [80], although it could be said this might reduce opportunities to utilise the uniqueness of lab teaching. The high rating for data collection and analysis goal items aligns with data management and handling as core bioscience skills [3] which practical labs can assist with [81].

The low ratings for labs generalising across multiple disciplines may be an intuitive reflection of education at advanced levels, but may have implications for students who pursue non‐bioscience careers postgraduation. Keeping good lab notebooks was also not highly valued. This skill is important for data collection in research labs but might be too niche for some teaching labs, given other goal priorities.

The quantitative results indicated that staff have comparable goals for Year 1 and 2 teaching labs, but as the questionnaire required participants to choose a year group, these findings rely on comparing between‐subject rather than more powerful within‐subject comparisons. There was still a near‐significant stronger focus on lecture connection and generalising across disciplines in first‐year labs, perhaps indicating that early‐year labs are more about reinforcing taught content and appreciating that Year 1 units are generally taken by a broader mix of honours and nonhonours students. Some differences emerged when participants had opportunities to compare year groups in interviews, discussed further in the student inquiry sections.

Teaching labs as a site of student contact was a prevalent interview theme, including connection with staff and other students, a chance for people to meet and work together. This can be especially valuable amidst what can be an isolating period [82]. Labs were also seen as a place for students to get hands‐on, gaining satisfaction from performing experiments themselves via physical interaction with equipment. The psychomotor domain is an understudied area of lab research but is thought to be a key component of authentic learning [83].

Staff perceive the hands‐on, active learning potential of labs [84] as key to developing necessary discipline‐specific and transferable skills for research and the future, in alignment with the recommendations and requirements of policy‐driven organisations [3, 4]. Students do desire hands‐on lab activities [85], and staff described being together in the lab and actively working as an enjoyable and largely unique opportunity for students.

### RQ2: Student inquiry

Staff provided insight into the levels of inquiry via the percentage of labs in which students must make decisions in different parts of the lab experience, whether the lab outcome was known and whether students could individualise aspects of the lab, adapted from [36, 39]. These responses were summed into an inquiry scale.

Elements of inquiry were common in teaching labs, although deeper implementation was unusual. On the whole, the inquiry levels in the rubric from Bruck *et al*. [36] mapped onto the level of adoption among this study's participants. Students analysing their own data was most common, then students determining their own method of analysis (Fig. 3). However, this study found that a choice of experimental procedure was rarer than a choice of problem to solve. This is a slight inversion from the higher levels of the rubric, which used choice of procedure to distinguish the two highest inquiry levels.

Those who used inquiry more showed significantly stronger goal focusses on certain goal items. Inquiry‐based learning gives students opportunities to engage in authentic investigation [27], and here this manifested as an increased focus on scientific reasoning, research experience and an appreciation for how science really works. Research is a collaborative endeavour, and higher inquiry staff had increased goal focus on communication and working together. Interestingly, the research skills item was not quite significant, but possibly this item's fairly generic wording left it open to participant interpretation. A specific example of a research‐lab skill (keeping a lab notebook) was considered significantly more important for higher inquiry staff. Other uncorrelated goals, such as understanding the usefulness of lab techniques and writing scientific reports, are equally applicable for inquiry‐based or cookbook‐style labs as they do not depend on whether protocols are provided for or produced by students. None of the goals were significantly negatively correlated with inquiry score, so perhaps staff who embed more inquiry activities are more enthusiastic about teaching labs generally and therefore rated more goals, more highly. Still, these findings offer insight into the specific goals staff who use inquiry are looking to achieve.

Students go through transition and development throughout their time at university [23], and questionnaire findings showed Year 2 students had significantly more individualisation of teaching labs than Year 1. This suggests that incorporating controlled aspects for students to consider, decide and tailor is the key feature differentiating the progression between Year 1 and Year 2 teaching labs, as opposed to broader experimental design decisions. This somewhat aligns with the QAA's recommendation for inquiry‐driven practicals prior to final‐year projects [3]. The progression aspect was prominently seen in the interviews too; teaching labs were perceived as tentative first steps to inquiry, especially for later years [30].

The term ‘inquiry’ was not dominant in participant discourse of teaching labs, although underlying concepts of student decisions and experimental design were. In interviews, teaching labs were seen as having the potential to be first steps for student inquiry. Many interviewees said they hoped or intended to incorporate more of these elements, referencing frustrations with ‘cookbook’ labs [86], but were often limited by the common concern of time constraints [28].

### RQ3: Teaching lab challenges

In questionnaires, staff were asked about enjoyment, sense of meaningfulness, time, budget and agency around teaching labs to determine whether any of these were challenges or constraints in realising goals and intentions for these sessions. Respondents had positive views of teaching labs, overwhelmingly considering them enjoyable and meaningful, which are both conducive to positive learning experiences [48, 87]. They believe their colleagues agree and that their students share similar views, if to a significantly more muted extent. This aligns with a previous study where staff demonstrated more negative expectations of students' feelings than students themselves did [49]. While still positive, the significantly reduced agreement for students may be an indication of some challenges in student‐centric practice contexts, such as anxiety and cognitive overload and limited prior lab experience. These stressors can inhibit student enjoyment and learning in labs [12].

Participants' mixed but overall slightly positive views on having sufficient time and agency for teaching lab decisions suggest a wide variety of practice contexts where many have the autonomy they need, others far less. Despite academia's high workload environment [46], teaching staff are generally finding (or making!) time to decide how to run their teaching labs. The broad agreement to budgetary constraints suggests a more systemic challenge; limited access to resources such as equipment or space could restrict staff achieving their teaching lab goals. Given the sizable investment of resources teaching labs require [8] in times of tightening university budgets [45], this may continue to be an issue. Given the significant association between sense of limited agency and belief that teaching labs would benefit from a bigger budget, it is possible that insufficient resources for teaching labs might restrict staff ability to design and run their preferred practical sessions. Despite perceptions of implementing inquiry learning as challenging and time‐consuming [27], these findings do not suggest that those who use greater inquiry are any more or less likely to find their labs enjoyable, meaningful or limited by these constraints.

The qualitative methods revealed issues of students' anxieties [52] and the wide disparity of prior lab experience [53], restricting their ability or willingness to prepare for, engage with and learn from these sessions. Staff recognised these were perennial, even increasing challenges, but addressed them with communication and human connection: by providing resources and information as clearly as possible, including online for asynchronous preparation [12] and encouraging reassuring dialogues with students.

### Limitations

The mixed‐methods combination of questionnaire and interviews utilised the strengths of both, but there were limitations to each. Questionnaire responses may have some self‐selection bias, if those who chose to participate were generally more invested in the teaching lab aspects of their role [88]. Statistical power was occasionally limited by the requirement for nonparametric tests [67], the ceiling effects seen in some questions and the smaller group sizes during comparisons (e.g. *N* = 34 and *N* = 33 for Years 1 and 2). Therefore, although many significant associations and differences were captured, others might have been missed.

The interviews in this study were shorter to encourage participation, but still allowed sufficient time to cover topics relevant to each research question. Of course, only so much depth can be reached in 30–45 min, so latent or personal aspects may have been left undiscussed. Furthermore, many related topics such as assessment, feedback, online learning technologies and more *were* discussed and considered during thematic analysis, but themes are not topic summaries [75], and not every aspect was explicitly covered in this qualitative interpretation.

### Recommendations for practitioners and future research

This study re‐emphasises the importance of active reflection, discussion and research about what makes teaching labs unique and special [8, 9, 10], and how best to use them for bioscience undergraduates.

Staff goals for teaching labs should be more explicitly discussed with internal colleagues and wider bioscience education communities. This more aligned picture could identify areas of teaching lab underutilisation, help with efforts to evaluate them in terms of overall curriculum objectives and create more cohesive, evidence‐based directions during teaching lab reforms [19, 35]. As staff participants identified here, this may include maximising the unique opportunities teaching labs bring for contact and connection with students and utilising their potential for active learning and skill development to reinforce theoretical sessions.

Understanding that inquiry can be introduced to varying degrees [34] before the final year [3] may help practitioners feel empowered to adopt it for themselves in a manner suitable for their students, gain experience and perhaps share their experiences to help others anticipate what to expect in a similar context. Staff participants here commonly adopted some elements of student inquiry and choice. They offered broadly positive views on it, although many share trepidations about student readiness and time investments, given contexts of high workload and competing priorities.

Interviewed staff recommended that student anxieties and underpreparedness around labs can be addressed via compassion, communication and connection, remaining aware of the skill disparity students are entering university with. Questionnaire participants perceived teaching lab budgets to be more limiting than sense of agency or time allotted for these sessions. Institutional budgets are challenging for individuals to address, especially in difficult financial times [45], but increased, concerted effort to produce evidence‐based practice for policymakers to recognise the unique potential of teaching labs may assist.

This research contribution could be extended in various ways. Continuing the mixed‐methods format, another round of questionnaires could quantitatively investigate interview themes such as perceptions of inquiry readiness or student lab anxieties, or ask directly for year group comparisons. Collecting additional details such as institution type, accreditation status and school/department size would enable comparison of perceptions across practice contexts. More systematic sampling strategies and larger samples would increase tests' statistical power, could be more representative and permit deeper inferential analysis.

Alternatively or additionally, running more in‐depth staff interviews could drill deeper into underlying reasons and more emotive aspects of the issues raised in this research. Incorporating other methods such as teaching lab observations or document analysis of lab manuals and outputs from policy, accreditation and regulatory bodies could reveal new dimensions when used alongside staff perceptions. As more educational research is done, we can gain a deeper understanding on how best to use these prevalent but resource‐intensive sessions to train the next generation of scientists and other professionals.

## Conflict of interest

This work is based on an Educational Research Master's dissertation at the University of Bristol. The Master's was partially funded by Learning Science Ltd (LearnSci), also the author's employer. LearnSci had no role in the author's choice of topic, methods or interpretation for this study. Some questionnaire participants may have known the author via use of LearnSci resources at their institution; however, interview participants were not directly known to the author via LearnSci to minimise role conflict.

### Peer review

The peer review history for this article is available at https://www.webofscience.com/api/gateway/wos/peer‐review/10.1002/2211‐5463.13687.

## Author contributions

EC conceived and conducted all aspects of the study, analysed the data and wrote the manuscript.

## Supporting information


**Table S1.** Questionnaire response demographics by A. gender, B. role type, C. employment types and D. time spent running, organising and/or managing teaching labs, by raw frequency (Freq.) and %. Summed percentages may not appear to total 100.0% due to rounding.
**Table S2.** Responses for: (A) total year group(s) respondents ran teaching labs for, (B) which year group they worked with most and (C) how many distinct teaching lab sessions they ran for that chosen year group.
**Table S3.** Kendall's tau correlation matrix for the 14 laboratory goal items. * = p < 0.05, **p < 0.001.
**Table S4.** Kendall's tau correlations for inquiry scale items (N = 79), *p < 0.05, **p < 0.001.
**Table S5.** Kendall's tau correlation matrix for perceptions of meaningful learning, enjoyment and constraints. *p < 0.05, **p < 0.001.
**Section S1.** Questionnaire questions.
**Section S2.** Example interview schedule.
**Section S3.** Quantitative supplementary figures.

## Data Availability

Available data are limited due to confidentiality agreements with participants. Contact the author for further information.
