# Supplementary material for: Staff goals, challenges, and use of student inquiry in undergraduate bioscience teaching laboratories
Source: FEBS Open Bio. 2023 Aug 28;13(10):1810–30. doi: 10.1002/2211-5463.13687 (PMC10760353; doi:10.1002/2211-5463.13687)

# Supporting Information

## Section S1: Questionnaire Questions

**Please confirm the statements below by checking the boxes.**

- I am a member of staff at a UK university and I have been involved in organising or running at least one bioscience teaching lab session for at least two academic years (including 2020/21 even if labs did not run)
- I have read the participant information above and agree to participate in this study.

**======================**

**Page 1 of 4**

In terms of undergraduate laboratory-based teaching / practical sessions, which student year group(s) do you work with? Select all that apply.

- Foundation Year
- Year 1
- Year 2
- Year 3+

From those you selected above, which student year group or groups do you work with the most? **Please consider only this year group for the rest of the questionnaire**.

- Foundation Year
- Year 1
- Year 2
- Year 3+

For this year group, how many teaching laboratory practicals do you help run, organise or manage? Each lab practical counts once even if it is run in multiple sessions across a year.

- 1 lab practical
- 2-3 lab practicals
- 4-9 lab practicals
- 10+ lab practicals

**======================**

**Page 2 of 4**

Below you will find a series of statements on the topic of goals for your teaching lab sessions, including experiments and activities. **Considering the year group you specified above**, and assuming in-person teaching labs in 2021/2022, how much do you agree with each of the following statements?

0 represents total disagreement, 100 represents total agreement.

| **Question as it appears in the questionnaire (order randomised)** | **Summary title for analysis and discussion** |
| --- | --- |
| Preparing students for research experience is a goal for my lab sessions. | Research Experience |
| My lab sessions are designed to encourage the development of scientific reasoning skills. | Scientific Reasoning |
| Understanding the usefulness of specific laboratory techniques is a goal for my lab sessions. | Laboratory Techniques |
| Students need to learn to work together in my lab sessions to succeed. | Working Together |
| My lab sessions are designed to develop a range of communication skills. | Communication Skills |
| My lab sessions are a place for students to learn to analyse data. | Analyse Data |
| Understanding the need for proper data collection techniques is a goal for my lab sessions. | Data Collection |
| There is a strong connection between lecture content and my lab sessions. | Lecture Connection |
| My lab sessions are designed to develop students’ mastery of lab techniques. | Technique Mastery |
| My lab sessions are designed to focus on skills that are transferable to research-oriented laboratories. | Research Skills |
| My lab sessions are designed to foster an appreciation for science in students. | Science Appreciation |
| My lab sessions are designed to generalise to multiple disciplines. | Generalise Disciplines |
| Teaching students how to write scientific reports is a goal for my lab sessions. | Scientific Reports |
| My lab sessions are designed to help students learn to keep a proper laboratory notebook. | Laboratory Notebook |
| This statement will be used to discard answers of people who are not reading the questions. Please select forty percent for this question. | - |

**What additional information would you offer about your laboratory goals?**

|  |
| --- |

**======================**

**Page 3 of 4**

**Considering the teaching lab sessions you run or organise for the year group you specified above, and assuming in-person teaching lab sessions in 2021/22, please complete the statements below.**

**For the year group specified above, in the next academic year:**

|  | **0 % of the time**  **1** | **1-25 % of the time**  **2** | **26-50 % of the time**  **3** | **51-75 % of the time**  **4** | **76-100% of the time**  **5** |
| --- | --- | --- | --- | --- | --- |
| I know what the experimental outcomes of laboratory sessions will be. |  |  |  |  |  |
| Students can individualise aspects of experiments (e.g. concentrations to use, species to study). |  |  |  |  |  |
| Students must determine the problem they want to solve in lab sessions. |  |  |  |  |  |
| Students have a choice of experimental procedures they want to use in a lab session. |  |  |  |  |  |
| Students must determine the method of analysis for themselves. |  |  |  |  |  |
| Students must analyse and interpret laboratory data themselves during/after a lab session. |  |  |  |  |  |

**Please indicate your level of agreement or disagreement with the following statements.**

|  | **Strongly disagree**  **1** | **Moderately disagree**  **2** | **Slightly disagree**  **3** | **Slightly agree**  **4** | **Moderately agree**  **5** | **Strongly agree**  **6** |
| --- | --- | --- | --- | --- | --- | --- |
| Teaching labs offer meaningful learning experiences for students. |  |  |  |  |  |  |
| My colleagues think teaching labs offer meaningful learning experiences for students. |  |  |  |  |  |  |
| Students think their teaching labs offer meaningful learning experiences. |  |  |  |  |  |  |
| I have limited agency to make decisions about teaching labs. |  |  |  |  |  |  |
| I have sufficient time to make decisions about teaching labs. |  |  |  |  |  |  |
| Teaching labs could be an enhanced learning experience if there was a bigger budget. |  |  |  |  |  |  |
| I enjoy the aspects of my job that involve teaching labs. |  |  |  |  |  |  |
| Students enjoy the aspects of their course that involve teaching laboratories. |  |  |  |  |  |  |

**======================**

**Page 4 of 4**

**For the year group selected above, in which topic area or areas are the teaching labs you organise or run? Tick as many as apply (or leave blank if you prefer not to answer).**

- Anatomy
- Biochemistry
- Bioinformatics
- Biology: plant science / zoology
- Cell and molecular biology
- Ecology / population biology
- Genetics
- Immunology
- Microbiology
- Physiology
- Other

**To which of the following do you most identify?**

- Male
- Female
- Non-binary / other
- Prefer not to say

**Which of the following best describes your role at your primary university?**

- Primarily teaching
- Primarily research
- Combined teaching and research
- Technical staff
- Other
- Prefer not to say

**Which of the following best describes your mode of employment?**

- Part time
- Full time
- Prefer not to say

**For how many years have you been involved in running, organising or managing teaching labs?**

- 2-3 years
- 4-5 years
- 6-10 years
- 11+ years
- Prefer not to say

You have reached the end of the questionnaire. Thank you for your participation.

This is a mixed-methods dissertation project, and I am seeking a small number of follow-up interviews (approximately 45 mins) to explore some perspectives on teaching labs in more depth. If you are interested in being contacted, please enter your email address into the box below.

*Please note this will allow me as researcher to identify your answers which will be used only to facilitate discussion in the interviews. Your data will never be published in a way where you could be identified. Entering your email does not guarantee you will be asked for interview, nor does it require you to participate if you are contacted.*

**Enter email (optional): ______________________**

**Please submit your responses using the 'Next' button below. Thank you again for your participation.**

## Section S2: Example Interview Schedule

**Pre-interview “checklist” - introductions and overview**

- Open Zoom meeting. Admit participant. Confirm audio and visuals working.
- Thanks for agreeing to participate
- Introduce myself, the dissertation project and reason for using interviews
- Describe semi-structured interview format (rough schedule, but flexibility around that)
- Outline recording policy as discussed in participant information
- Check for questions before start recording
- Start Recording

**NB: the following are sample questions or prompts only, as the interview was semi-structured**

**Section 1: General questions about teaching labs and your role**

- To start with, please could you tell me the ways in which you are involved in bioscience teaching labs?
- The *[number specified in questionnaire]* labs that you run for *[Year X]* students, are they all in the same module?
- What topics are covered?
- How many students in each lab session?
- How long are the sessions?
- Did you design the lab(s), or was it passed to you from another colleague?
- Do they change much year on year?

*Further inquire into aspects they brought up themselves e.g. pre-lab tasks, assessment, recent changes etc, even if listed below, for a more natural flow.*

**Section 2: Goals for teaching labs**

Outline / refresh memory of the goals list, and which year group they focused on

- So, just looking down the list, your top-rated goals included those about [goal item]. Can you tell me more about that? Give an example?

*Repeat for different items if suitable*

- You chose lower scores on goals related to **[goal item].** Can you tell me more about that?

*Repeat for different items if suitable*

- Your response in the free-text box was about *[topic or topics mentioned]*. Please could you tell me more about that?
- Do you have different goals for the [other year(s) worked with] students compared to the [main year]?
- Do students in different year groups approach labs differently?

**Section 3: Inquiry-based learning**

Remember, do not use “inquiry” terminology unless participant raises it first

- Are there any aspects of your labs which students individualise or make experimental decisions about? If yes, can you give an example?
- How does this go down with students?
- You said that in *[% from questionnaire]* of your labs, students *[use X aspect of inquiry]* – can you tell some more about this? Give an example?
- Was this something you introduced, or inherited from previous staff?
- How does this go down with students?

*Repeat for different items if suitable*

- Is there is a difference in the level of choice *[main year group]* and *[other year group(s)]* students get. If so, in what way? Why?

**Section 4: Enjoyment and meaningful learning**

- What aspects do you think **students** enjoy about teaching labs, compared to other forms of learning like lectures/workshops/tutorial?
- Any aspects they find less enjoyable, challenging or frustrating?
- What aspects of teaching labs do **you** particularly enjoy?
- Any aspects you find less enjoyable, challenging or frustrating?
- You *[level of agreement]* with the statement that teaching labs offer meaningful learning - can you discuss this,

**Section 5: Change and constraints**

The last section is about design and change within teaching labs

- If there was a change you wanted to make to a teaching lab for next academic year, what would the process be like? Who would be involved?
- Is there anything in mind that you might change?
- You *[level of (dis)agreement]* that you had sufficient time to make decisions about teaching labs – could you tell me more about that?
- You *[level of (dis)agreement]* that you had limited agency in making decisions about teaching labs – could you tell me more about that?
- You *[level of (dis)agreement]* that teaching labs could be an enhanced learning experience if there was a bigger budget – could you tell me more about that?

Finally, a few questions on the unusual year we’ve had and the future

- How were teaching labs affected by the lockdown restrictions of 2020/2021?
- What aspects will you be keen to return to how they were?
- Are there any lessons learned / things you might keep, moving forward?

**Post-interview “checklist” - wrapping up, debrief and next steps**

- “Those are all the questions I had. Is there anything else you would like to add, or are there any areas you would like to return to?” (If yes: address these. if no: proceed.)
- Stop Recording
- Thank participant for their time and their answers
- Discuss next steps: Zoom will provide recording after the meeting closes. I will send link, if wish to add, amend or withdraw in next ten days, get in touch.
- Any final questions?
- Close meeting

## Section S3: Quantitative Supplementary Figures

**Table S1**. Questionnaire response demographics by: **A**. gender, **B**. role type, **C**. employment types and **D**. time spent running, organising and/or managing teaching labs, by raw frequency (Freq.) and %. Summed percentages may not appear to total 100.0% due to rounding.


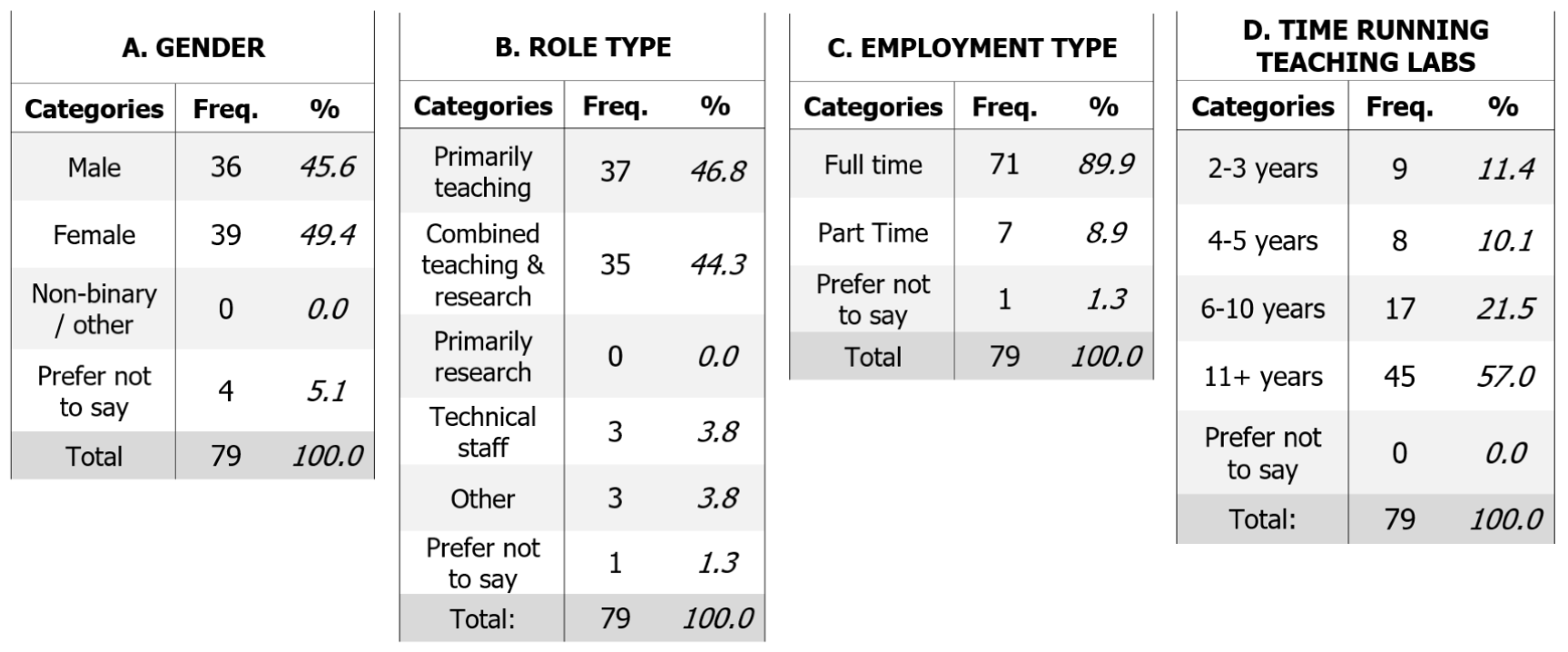


**Table S2**. Responses for: (**A**) total year group(s) respondents ran teaching labs for, (**B**) which year group they worked with most, and (**C**) how many distinct teaching lab sessions they ran for that chosen year group.


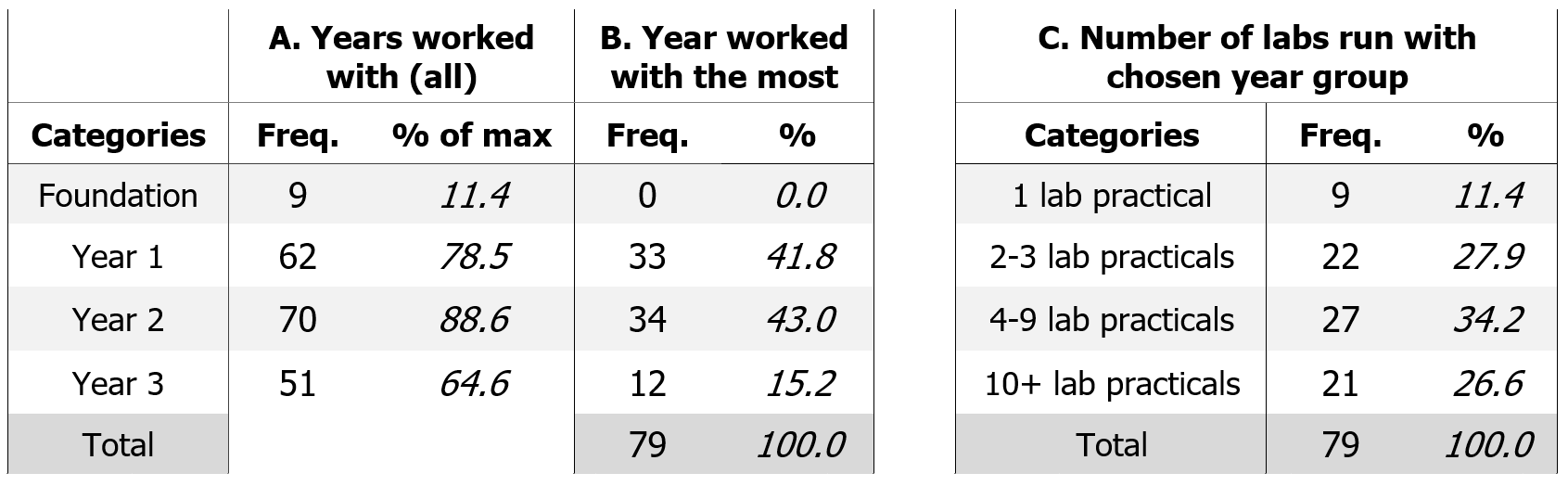


**Table S3**. Kendall’s tau correlation matrix for the 14 laboratory goal items. *=p<.05, **p<.001.


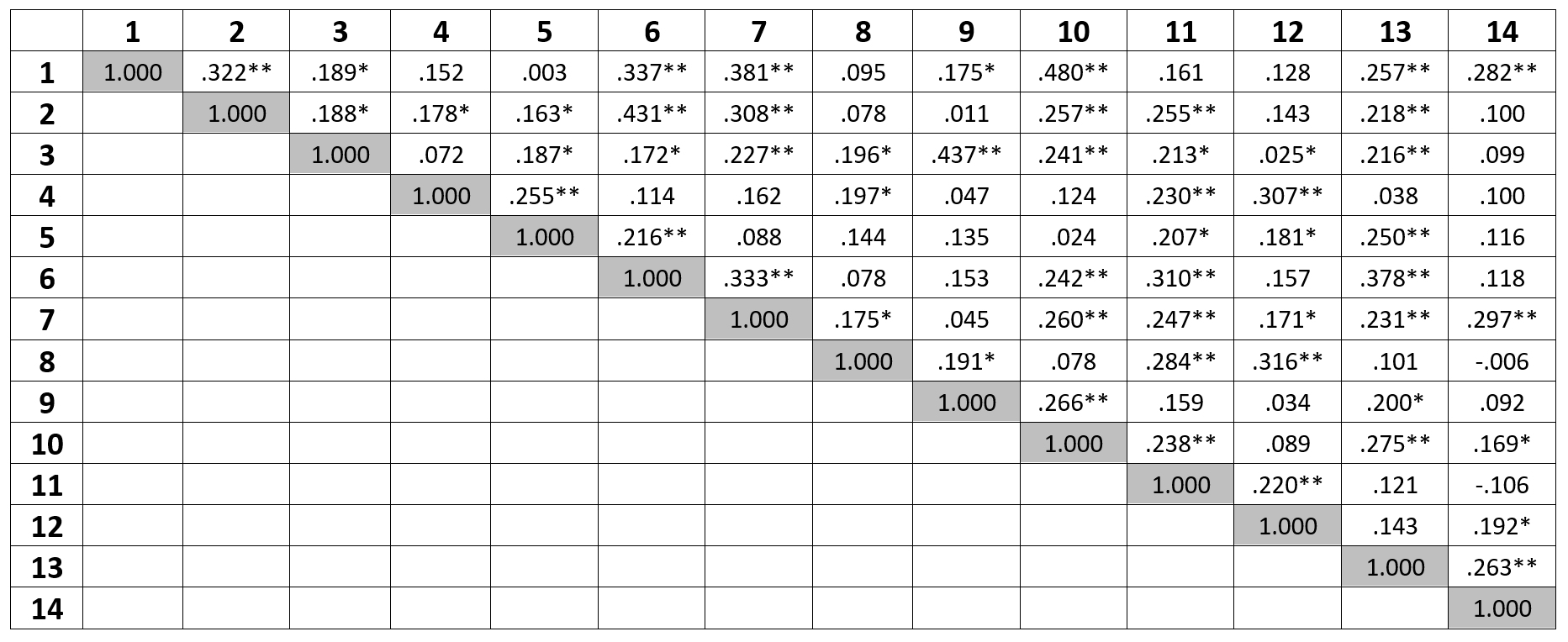


**Table S4.** Kendall’s tau correlations for inquiry scale items (N=79), *p<.05, **p<.001.


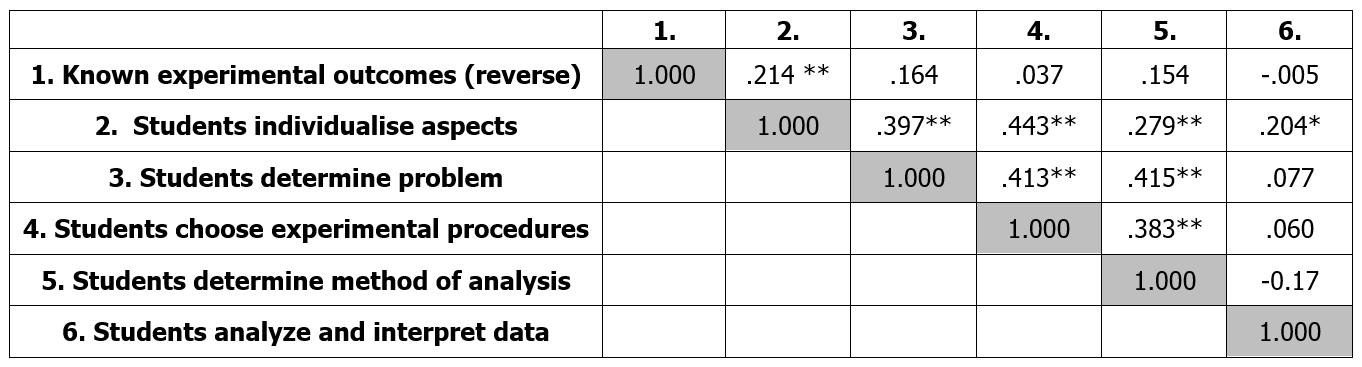


**Table S5**. Kendall’s tau correlation matrix for perceptions of meaningful learning, enjoyment and constraints. *p<.05, **p<.001.


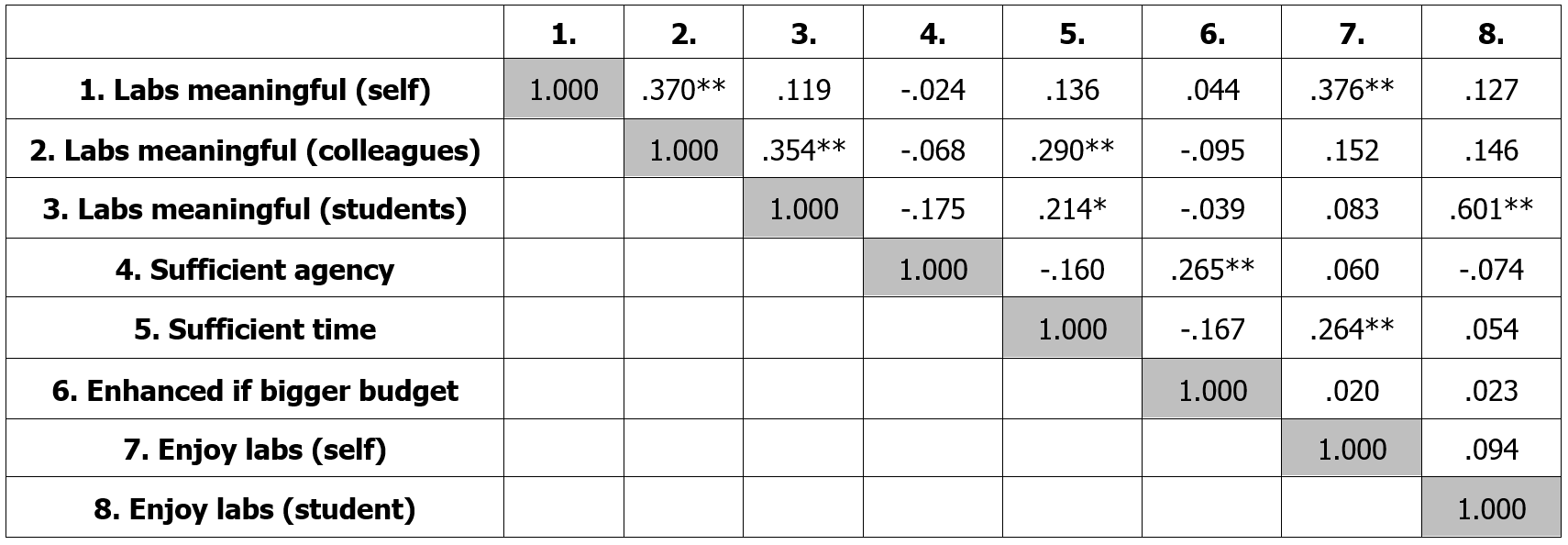

Supplement: Supplementary file 1 — Table S1. Questionnaire response demographics by A. gender, B. role type, C. employment types and D. time spent running, organising and/or managing teaching labs, by raw frequency (Freq.) and %. Summed percentages may not appear to total 100.0% due to rounding. Table S2. Responses for: (A) total year group(s) respondents ran teaching labs for, (B) which year group they worked with most and (C) how many distinct teaching lab sessions they ran for that chosen year group. Table S3. Kendall's tau correlation matrix for the 14 laboratory goal items. * = p < 0.05, **p < 0.001. Table S4. Kendall's tau correlations for inquiry scale items (N = 79), *p < 0.05, **p < 0.001. Table S5. Kendall's tau correlation matrix for perceptions of meaningful learning, enjoyment and constraints. *p < 0.05, **p < 0.001. Section S1. Questionnaire questions. Section S2. Example interview schedule. Section S3. Quantitative supplementary figures. [file FEB4-13-1810-s001.docx]
